# INTERPLAY BETWEEN LOSS OF PROTEOSTASIS AND CELLULAR SENESCENCE: A SPOTLIGHT ON MISFOLDED PROTEIN QUALITY CONTROL

**DOI:** 10.1093/geroni/igac059.1666

**Published:** 2022-12-20

**Authors:** Rahul Samant

**Affiliations:** Babraham Institute, Cambridge, England, United Kingdom

## Abstract

Loss of protein homeostasis (‘proteostasis’) and onset of cellular senescence are two conserved hallmarks of ageing. Healthy proteostasis relies on tightly-regulated intracellular quality control circuits that co-ordinate clearance of potentially toxic misfolded proteins arising from various internal or external stresses throughout an organism’s lifespan. Proteostasis imbalances are mechanistically linked to a broad range of ageing-associated diseases, and are also characteristic of cellular senescence—a permanent cell cycle arrest that prevents uncontrolled proliferation during development, injury repair, and tumorigenesis, but drives ageing-associated frailty, degeneration, and therapy resistance. Across a range of replicative and stress-induced senescence models in primary human cells, we have discovered differences in how misfolded proteins are triaged when compared with proliferating, quiescent, or immortalised cells—especially at the level of ubiquitin-mediated protein clearance systems. Given recent findings that proteostasis modulators act as senolytics with geroprotective properties, our work highlights the need for an improved fundamental understanding of how different ageing hallmarks are inter-connected in order to drive advances in human healthspan.

